# Comparative Study on the Distribution of Essential, Non-Essential Toxic, and Other Elements across Trophic Levels in Various Edible Aquatic Organisms in Sri Lanka and Dietary Human Risk Assessment

**DOI:** 10.3390/toxics10100585

**Published:** 2022-10-04

**Authors:** Anura Upasanta-Kumara Wickrama-Arachchige, Keerthi S. Guruge, Hinako Tani, Tilak Siri Dharmaratne, Marappullige P. Kumara, Yasuaki Niizuma, Takeshi Ohura

**Affiliations:** 1 Faculty of Fisheries and Ocean Sciences, Ocean University of Sri Lanka, Tangalle HB 82200, Sri Lanka; 2 Division of Hygiene Management Research, National Institute of Animal Health, National Agriculture and Food Research Organization, Tsukuba 305-0856, Japan; 3 Graduate School of Veterinary Science, Osaka Metropolitan University, Osaka 598-8531, Japan; 4 Graduate School of Agriculture, Meijo University, Nagoya 468-8502, Japan; 5 Gem and Jewelry Research and Training Institute, Kaduwela CO 10115, Sri Lanka

**Keywords:** toxic metals, trophic level, mercury, aquatic organisms, tuna, health risks

## Abstract

Thirty-six elements are categorized as essential but toxic in excess amount (EBTEs), non-essential toxic (NETs), and Other in 29 different edible aquatic species dwelling in offshore pelagic, and coastal and estuarine (CE) ecosystems were investigated in Sri Lanka. Elements were analyzed using an energy-dispersive X-ray fluorescence (EDXRF) spectrometer, and an NIC MA-3000 Mercury Analyzer. EBTEs showed a negative relationship, whereas NETs showed a positive relationship between the concentration (mg/kg wet weight) and trophic levels in both ecosystems. EBTEs showed trophic dilution, whereas NETs showed trophic magnification. Some elements in a few organisms exceeded the maximum allowable limit which is safe for human consumption. There was a positive relationship (R^2^ = 0.85) between the concentration of mercury and body weight of yellowfin tuna (YFT). For the widely consumed YFT, the calculated hazard index (HI) for the non-carcinogenic health and exposure daily intake of NETs for adults were 0.27 and 9.38 × 10^−5^ mg/kg bw/day, respectively. The estimated provisional tolerable weekly intake (PTWI) (μg/kg bw/w) was 0.47 for arsenic and 0.05 for antimony, cadmium, mercury, and lead. The HI and PTWI values were below the recommended limits; thus, consumption of YFT does not pose any health risk for Sri Lankan adults.

## 1. Introduction

Fish and other aquatic species rich in proteins and other essential nutrients are considered as healthy human food; thus, they have been included in the daily diet of many of the world population [1]. Direct use of fish for human consumption has been increasing in the past decades, showing an average of slightly higher than 20.5 kg year^−1^ for each of the world’s inhabitants in 2018 [2]. It is nearly 16.6 kg year^−1^ for all sectors in Sri Lankans [3]. Fish consumption has been popular among the world’s human population, and there has been a significant rise in global fish production for direct human consumption during the last decades [2]. In the light of the increased demand for aquatic species as human food, the quality and safety of food products have been essential for many countries [4]. However, there is a growing consumer concern over the quality (e.g., nutritional value and freshness) and safety (e.g., contamination with hazardous and bioaccumulative chemicals) of fish and fish products. Thus, the nutritional quality of the aquatic species is hindered by the safety deterioration of such hazardous elements, including some metals. 

The elements, including metals, are classified into several categories. Some of the elements are essential but toxic in excess quantities (EBTEs) of such elements to humans and fish (e.g., tin (Sn), ferrous (Fe), copper (Cu), chromium (Cr), zinc (Zn), and selenium (Se)). Some of these toxic and essential elements have been reported in herbal medicinal products [5], in snacks [6], and aquatic organisms including fish [7,8]. A certain level of some elements that exceed the maximum allowable limit (MAL) in the intake are non-essential and toxic (NETs) to humans (e.g., arsenic (As), antimony (Sb), cadmium (Cd), mercury (Hg), and lead (Pb)) [9,10]; however, there are several other elements, including some metals (Others), which their specific biological functions are not well known. In this study, we hypothesized that certain toxic elements might be accumulated in larger quantities in the tissues of aquatic organisms at higher trophic levels (TLs). There are various routes of entering these elements into organisms’ bodies. Anthropogenic interventions, such as industrial inputs [11], agricultural waste [12], and natural origins, such as underwater volcanic eruptions [13], can permit toxic elements to enter aquatic ecosystems that make a route to enter into the body of the aquatic animals, ultimately accumulating them in their consumers, such as humans.

Fishes play an essential role in the aquatic food web that represents several TLs [14]. They are also useful in monitoring the contamination of heavy metals in aquatic ecosystems as a biological indicator [15]. Several chronic and negative health effects on humans from consuming contaminated fish with toxic metals have been reported [16]. The uptake rates of elements by a fish are governed by the chemical and physical state of the elements, as well as the environmental and species-specific biological factors [17]. Thus, the analysis of elements in different aquatic species living in different environments, such as the offshore pelagic (OP) ecosystem, which is away from the mainland, coastal, and estuarine ecosystem (CE), which is near to mainland, would provide an insightful understanding of the distribution of these elements in the tissues of edible aquatic species. Moreover, Canli & Atli [18] reported fish-specific and tissue-specific disparities in the accumulation of different elements in fish bodies. There are several previous studies, in particular, analyses of mercury in fish in Sri Lanka (e.g., [9,19,20,21,22,23,24,25]). Temporal and spatial monitoring of such an element in the case of the concentrations in tissues is essential in identifying current and future trends, especially for widely consumed aquatic species, such as yellowfin tuna (YFT) (Scientific name: *Thunnus albacares*) in Sri Lanka. Rathnasuriya et al. [16] reported that coastal communities that consume a considerably higher quantity of fish mainly, including YFT, contain elevated concentrations of Hg in hair, directing the need for further assessment of human health risks of toxic elements. 

There are many analytical techniques used for quantitative analysis of elements. Some techniques include mass spectrometry techniques and X-ray microanalytical techniques for analyzing metallic elements in biological samples [26]. The distribution of elements is also analyzed using the atomic absorption spectrophotometry [24,27,28], inductively coupled plasma mass spectrometry [29], and EDXRF spectrometry [30]. Comparatively, the EDXRF rapidly quantifies elements and has accurate, low-cost results; therefore, in this study we used the EDXRF technique, with NIC MA 3000 mercury analyzer which is highly sensitive for detection of Hg. 

The objectives of this study are to (1) evaluate 35 elements giving special reference to toxic elements classified into EBTEs and NETs present in tissues of 29 edible aquatic species, (2) compare the concentrations of elements between different TLs considering two different marine areas in Sri Lanka, (3) evaluate MALs of each EBTE and NET in different aquatic species, (4) compare the concentrations of elements between dark and white muscles of two different tuna species (i.e., YFT and Skipjack tuna (SJT) (Scientific name: *Katsuwonus pelamis*), and (5) evaluate bioaccumulate potential of Hg in YFT and human health risk of ingestion of Hg by consuming YFT. Our primary objective is to analyze the maximum number of elements, including toxic metals, in a broad range of species representing different TLs. Therefore, we used one specimen from large individuals or several individuals for small organisms pooled into one homogenized sample. Fish are considered as a potentially accessible and comparatively cheap source for obtaining protein that is needed for humans, so there is a contemporary trend of consuming a variety of fish. Therefore, the amount of EBTEs and NETS stored in such a diet should be studied for the preparation of dietary guidelines for safe consumption of fish. To the best of our knowledge, this is the first report that presents TLs-associated accumulation patterns of elements in wide-ranging aquatic edible species, especially in Sri Lanka, which gives important data for preparing dietary guidelines.

## 2. Materials and Methods

### 2.1. Sample Collection

Eighty individual organisms representing 36 different fresh samples from 29 different species were collected from the local fish market in Sri Lanka, representing edible aquatic organisms dwelling in the OP and CE ecosystems around Sri Lanka (Table S1). They were the most consumed aquatic organisms by Sri Lankans. There were 18 species of finfishes (15 species of bony fishes, one species of ray, and one species of shark), shellfishes (one species of squid) in OP, and 11 species (ten species of finfishes and one species of shrimp) in CE. The TLs of the organisms were assumed by their different feeding habits (Table S1), and some of them were previously analyzed for halogenated polycyclic aromatic hydrocarbons (HPAHs) (e.g., [14,31]). In the OP samples, there were two different kinds of tuna species: YFT (*n* = 7) and SJT (*n* = 3). They were selected to determine species-specific and muscle-specific (i.e., white muscle versus red muscles) concentration variations of target compounds. Moreover, YFT with different body masses, including small, medium, and large individuals, were used to analyze the bioaccumulation of target elements. One sample from one specimen was randomly selected for large organisms, whereas several replicates of tuna samples were analyzed, and several individuals from small-sized organisms were pooled to prepare homogenized samples. This strategy was implemented to cover a wide range of biota representing many species [14]. Sri Lankans consume comparatively large quantities of aquatic organisms to obtain their protein requirements, and the selected species were the most popular edible species in their diets. The feeding habitat and living environmental conditions of the studied fauna are given in Supplementary Table S1. Here the fork lengths and somatic body weights were also recorded if the entire specimens were available. Samples were preserved in ice after washing them with distilled water before being transported to the laboratory. The head, fins, and viscera were removed from small to medium-sized specimens, and approximately 200 g of muscle sample was taken from large fishes from the anterior dorsal region near the pectorals. These samples were stored in a freezer below −40 °C until sample preparation for analysis.

### 2.2. Toxic and Other Elements 

This study considered three types of elements based on toxicity, as stipulated by Bosch et al. [10]. Toxic elements were grouped into two categories: EBTEs and NETs. The remaining elements were included in the third group: 3) Other. The elements that included some metals in EBTEs were Sn, Fe, Cu, Cr, and Zn; NETs were As, Sb, Cd, Hg, and Pb; Others were nickel (Ni), magnesium (Mg), aluminum (Al), silicon (Si), phosphorus (P), sulfur (S), chlorine (Cl), potassium (K), calcium (Ca), titanium (Ti), manganese (Mn), cobalt (Co), selenium (Se), bromine (Br), rubidium (Rb), strontium (Sr), tellurium (Te), iodine (I), hafnium (Hf), tantalum (Ta), platinum (Pt), gold (Au), gallium (Ga), germanium (Ge), and yttrium (Y). However, none of these samples detected Ga, Ge, and Y, and they were excluded from the discussion. Wet weight (ww) base concentrations (mg/kg) of the elements in fish samples were calculated. 

### 2.3. Sample Preparation and Analysis

All samples were freeze-dried to a constant weight, while white and dark muscles were separated from each transverse section of YFT and SJT. All the samples were homogenized to a fine powder. Nearly 2 g of the sample and 400 mg of the binder (Spectro Blend, Chemplex Industries, INC, Palm City, FL, USA, 44-µm powder; blending, grinding, and brightening additive; chemical composition: 81% C, 13.5% H, 2.9% O, and 2.6% N) precisely weighted and mixed with a natural stone mortar and pestle. The mortar and pestle were cleaned with methanol three times before use. The mixed sample was loaded to the pellet machine, where 800 pa load pressure was applied for 1 min to prepare the pellet. The created pellet was covered with tinfoil and stored in a freezer for analysis. The pelleted samples were analyzed using Rigaku EDXL-300 energy-dispersive X-ray fluorescence (EDXRF) spectrometer, Japan. The equipment was calibrated using the reference samples before use. The detection limit of the EDXRF is 1.0 mg/kg for all compounds, so half of the detection limit (0.5 mg/kg) was used for analysis by considering the maximum risk for all concentration values of elements that were below the detection limit (<1.0 mg/kg dry weight). Additionally, mercury was analyzed separately using NIC MA-3000 Mercury Analyzer, Japan. Approximately 5 mg of each homogenized sample was accurately measured with an analytical balance. The measured samples were placed in ceramic-coated bath containers and then kept in trays. The equipment was calibrated before use, and two replicates from one sample were analyzed. 

### 2.4. Stable Isotope Analysis

Stable isotope analysis was performed using delipidated samples [14]. Organic solvents were used to remove lipids from samples. Then, pellets were prepared for each sample using 0.45 to 0.55 mg of homogenized delipidated samples placed in tin capsules. Three pellets were made from one sample, and all the samples were analyzed using a continuous-flow isotope ratio mass spectrometer (ANCA-GSL and Hydra 20–20, Sercon Ltd., Cheshire, UK) at the stable isotope facility of Meijo University, Japan. The mean stable isotopic value for the three replicates of samples was obtained as ratios, and they were expressed in δ notation as parts per thousand (‰) deviation from the Pee Dee Belemnite for 13C and atmospheric N_2_ for 15N [32].

δX = [(Rsample/Rstandard) − 1] × 1000 (1)

Here, X is 13C or 15N, and R is the isotopic value (13C/12C or 15N/14N). A consumer in one TL shows, on average, a 3.4% increase in δ15N relative to the prey they consumed in the immediately lower TL and, the primary carbon source of the food in different TLs are determined by the δ13C, which shows a 1% increase with each TL [32]. 

Different environmental conditions in different aquatic ecosystems permit different stable isotopic signatures among organisms. Nitrogen can be readily enriched in a CE near land by an anthropogenic source (e.g., agricultural waste, fertilizers, and sewage) and river run-off consisting of inorganic nutrients; thus, causing the 15N enrichment of particulate organic matter [33] and showing comparatively higher δ15N values in the tissue samples of aquatic animals living in the ecosystem [34]. Some of the physical parameters, such as temperature and salinity fluctuations, are more pronounced in CE than that in such parameters in an offshore environment, and these changes are influenced by changing δ15N [14,35]. Wickrama-Arachchige et al. [14] found different δ15N and δ13C signatures between the aquatic organisms sampled in OP and CE. As there was spatial variability in δ15N and δ13C values [14,35], we separately evaluated the stable isotope signatures in these edible aquatic organisms sampled in the OP and the CE in this study. 

### 2.5. Statistical Analysis

The total concentration of the three types of elements (EBTEs, NETs, and other) was evaluated between TLs of the samples collected at OP and CE using one-way ANOVA. The concentrations of the target compounds between white and dark muscles of YFT and SJT were tested using the two-sample *t*-test. Linear regression was separately executed to evaluate any significant relationship between the concentrations of toxic elements (EBTEs and NETs) and body weight of YFT by considering very large, medium, and small body weights. Normal distribution and homogeneity of variance were investigated before the parametric analysis to maintain the level of significance as *p* < 0.05. All the statistical analyses were performed with MINITAB 19, and the graphs were prepared using Microsoft Excel 2019 and MINITAB 19 statistical software. 

### 2.6. Health Risk Assessment

#### 2.6.1. Estimated Daily Intake (EDI) of *T. albacares* (YFT)

The estimated daily intake (EDI) of *T. albacares* by Sri Lankans is calculated using the following equation [9].

(2) $$\mathrm{EDI}=\left[\mathrm{NET}\right]\times \left[\frac{\mathrm{DFC}yft-\mathrm{sl}}{\mathrm{BW}}\right]$$

Here, [NET] is the mean Hg concentration in YFT muscle in wet weight (mg/kg ww); DFC*yft*-sl denotes the daily per capita fish consumption of YFT in Sri Lanka (g/day), and it was 3.8 g/day (0.0038 kg/day) for both genders assuming that the eating patterns are similar between males and females [9]; BW (kg) represents the average body weight, and average adults’ BW was 60 kg [36].

Mean values of each NET were calculated from YFT to obtain the total concentration of [NET]. Human health risk assessment of Hg exposure was performed assuming all the Hg in YFT are present in methyl mercury (M-Hg) [9]. Some studies reported that the total Hg level in fish was identical to the M-Hg level [37]; however, Razavi et al. [38] and Strandberg et al. [39] reported that M-Hg in fish muscles is about 90% of the total Hg. Jinadasa et al. [9] also assumed that ratio followed the same method for better comparisons in this study. 

#### 2.6.2. Target Hazard Quotient (THQ) of Consuming *T. albacares* (YFT)

The non-carcinogenic health of YFT fish consumers was estimated by calculating target hazard quotient (THQ) values given in Equation (3) [9,40,41]. The USEPA Region III risk-based concentration table was referred to obtain the oral reference dose (RfD).

(3) $${\mathrm{THQ}}_{i}=\frac{\mathrm{EF}\times \mathrm{ED}\times \mathrm{FIR}\times \left[{\mathrm{NET}}_{i}\right]}{\mathrm{BW}\times \mathrm{ATn}\times {\mathrm{RfD}}_{i}}\times 10^{-3}$$

Here, THQ*_i_* = target hazard quotient of *i*th NETs; EF = exposure frequency is 365 days/year; ED = exposure duration is 70 years for non-cancer risk as used by USEPA [42]; FIR = fish ingestion rate is DFC*yft*-sl, which is (3.8 g/day); [NET*_i_*] = concentration of *i*th non-essential toxic element in fish muscles (mg/kg); BW = average body weight (kg); ATn = average exposure time for non-carcinogens (EF × ED), as used by Keshavarzi et al. [40] in characterizing non-cancer risk. RfD = reference dose of *i*th toxic element (3.0 × 10^−4^, 3.5 × 10^−4^, 1.0 × 10^−3^, and 4.0 × 10^−3^ for Hg, As, Sb, Cd, and Pb, respectively) mg/kg/day. There will be a potential human health risk by consuming the contaminated food if THQ ≥ 1 [43].

#### 2.6.3. Hazard Index (HI)

The HI was employed to estimate the human health risk of multiple toxic elements [44] based on the assumption of additive effects from a mixture of toxic elements in YFT muscles using the following equation:

(4) $$\mathrm{HI}=\sum_{i}^{n}\mathrm{THQ}$$

Here, the total number of considered NETs is denoted as *i*th to *n*th. There is a public health concern about consuming the particular aquatic species if the HI values exceed the limits, which is the overall exposure above 1 calculated using THQs is likely to result in chronic risk, causing adverse health effects to lifetime exposure of human, and the HI below 1 is normally be considered as acceptable [43,45]. 

#### 2.6.4. Provisional Tolerable Weekly Intake (PTWI) for *T. albacares* (YFT)

The PTWI of individual NETs by consuming YFT was estimated using Equation (5) [46].

(5) $${\mathrm{PTWI}}_{i}=\frac{\left[{\mathrm{NET}}_{i}\right]\times \mathrm{DFC}yft-\mathrm{sl}\times 7}{\mathrm{BW}}$$

Here, PTWI*i* is the provisional tolerable weekly intake of the “*i*th” element, and NET*i* is the concentration of “*i*th” non-essential toxic element. There are different reference PTWI values for Hg and As set by different international organizations. For Hg, they were 5 μg/kg body weight per week (bw/w) for Hg [47] and 0.0033 mg/kg of body weight for M-Hg [48], and 1 mg/kg (M-Hg) is an action level for methylmercury used by the US Food and Drug Administration [49]. The PTWI values for total As were 3000 μg/kg bw/w [47,50] and 15 μg/kg bw/w for inorganic As [48]. The PTWI values for Cd and Pb were 7 μg/kg bw/w [50,51] and 25 μg/kg bw/w [47,50], respectively. The tolerable daily intake for Sb was 360 μg/d [52,53].

## 3. Results

### 3.1. Concentrations and TLs vise Accumulation of Elements

**Figure 1:**
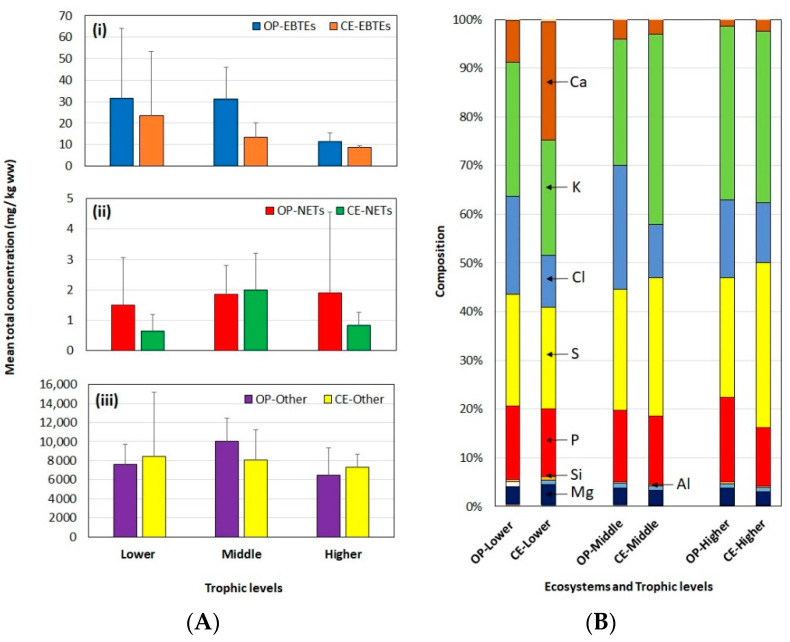
Mean total concentrations + standard deviation (SD) of elements (mg/kg ww) (**A**) and percentage compositions of each element between trophic levels (**B**) of the different edible aquatic organisms collected in offshore pelagic (OP) and coastal and estuarine (CE) ecosystems in Sri Lanka. OP: offshore pelagic, CE: coastal and estuarine; EBTEs: essential but toxic in excess amount (**i**), NETs: non-essential toxic (**ii**), and Other: other elements (**iii**).

**Figure 2:**
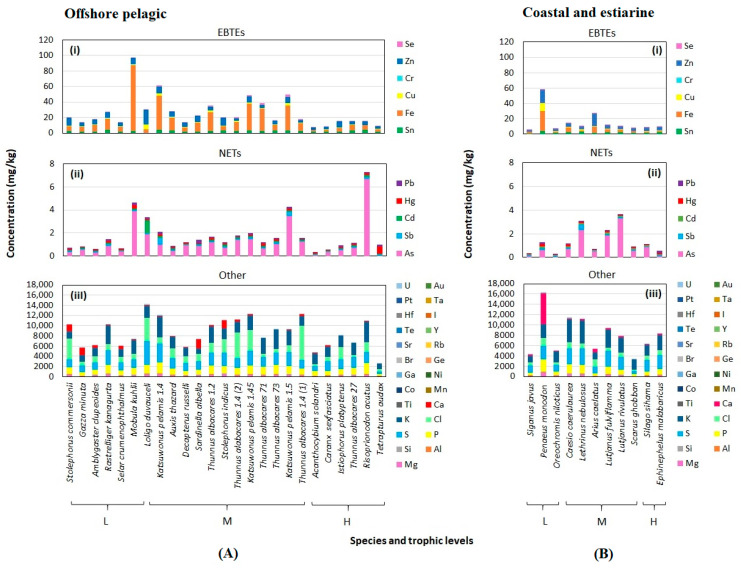
Concentrations of elements (mg/kg ww) are categorized into three groups: EBTEs: essential but toxic in excess quantities (**i**), NETs: non-essential toxic (**ii**), and Other (**iii**) among edible aquatic organisms collected in offshore pelagic (**A**) and coastal and estuarine (**B**) ecosystems in Sri Lanka. L = lower trophic level, M = middle trophic level, and H = higher trophic level.

For the OP organisms, the concentration of EBTEs was Cr < Se < Cu < Sn < Zn < Fe, while that of NETs was Pb < Sb < Cd < Hg < As, and that of the most abundant other elements was Sr < Br < Si < Al < Mg < Ca < P < Cl < S < K (Table S2). For the CE organisms, the concentration of EBTEs was Cr < Se < Cu < Sn < Fe < Zn, while that of NETs was Cd < Hg < Pb < Sb < As, and that of the other elements was Br < Sr < Si < Al < Mg < Ca < Cl < P < S < K (Table S3). The stable isotope analysis revealed that there were three possible trophic levels: lower TL (LTL), middle TL (MTL), and higher TL (HTL) (Figure S1). The most abundant elements in every TL were K, S, Cl, P, Ca, and Mg (Figure 1). However, in each TL, some organisms showed elevated levels of total element concentrations (Figure 2). Composition showed that the level of Ca diminished from lower to higher TLs; however, the distribution of other elements appears to be the same between TLs (Figure 1B).

TL associated analysis of the elements in the OP aquatic organisms showed possible biomagnification of NETs while there was possible biodilution of EBTEs and other elements (Figure S2A). The mean concentrations of total EBTEs were 31.6, 31.0, and 11.4 mg/kg for LTL, MTL, and HTL, respectively, demonstrating the following magnitude order: HTL < MTL < LTL (Figure 1Ai). There was a significant difference in the mean EBTEs in the OP between TLs (Kruskal–Wallis: H = 10.65, DF = 2, *p* = 0.005) (Figure S2Ai). Furthermore, the concentration of EBTEs had a negative correlation (R^2^ = 0.037) with δ15N, showing that EBTE biodilute in the food web may be because these essential elements may be readily used as nutrients for building their bodies and maintaining their health in the higher TL organisms (Figure S2Ai). The mean concentrations of total NETs were 1.49, 1.86, and 1.89 mg/kg for LTL, MTL, and HTL, respectively (Figure 1Aii). Although there were no significant differences in total NETs between TLs, the mean concentration of these elements showed a gradual increase in their concentrations with the increase in TLs in the OP (LTL < MTL < HTL) (Figure 1Aii). Moreover, there was a positive correlation (R^2^ = 0.03) between the concentration of NETs and δ15N values of the OP organisms, indicating possible biomagnification (Figure S2Aii). These results cope with our hypothesis that the tissue concentration of several NETs increases with increasing TLs may be because of bioaccumulation. The mean concentrations of the other elements in the OP were 7610.2, 10,012.3, and 6503.8 mg/kg for LTL, MTL, and HTL, respectively; however, the differences was significant between TLs (Kruskal–Wallis: H = 6.66, DF = 2, *p* = 0.036), showing the concentration increment of HTL < LTL < MTL (Figure 1Aiii). There was a biodilution of other elements in the OP organisms, where there was a negative correlation (R^2^ = 0.009) between the concentrations of other elements and δ15N values (Figure S2Aiii). Relatively larger fishes grouped in the HTL need to consume lots of nutrients for their body maintenance so that they might use other elements effectively and efficiently. It may also be the reason for low concentrations of Other and EBTEs in the muscles of the animal belonging to HTL. The same kind of trend was shown by the CE organisms.

The CE aquatic organisms also showed possible biomagnification of NETs and possible biodilution of EBTEs and Other elements (Figure S2B). The mean concentrations of total EBTEs were 23.6, 13.5, and 8.82 mg/kg for LTL, MTL, and HTL, respectively, showing the following magnitude order: HTL < MTL < LTL (Figure 1Ai). These differences were not statistically significant (*p* > 0.05). However, the concentration of EBTEs showed a negative correlation (R^2^ = 0.11) with δ15N values, suggesting that EBTEs biodilute in the CE food web (Figure S2Bi). The mean concentrations of NETs were 0.64, 1.99, and 0.84 mg/kg for LTL, MTL, and HTL, respectively, showing the following magnitude order: LTL< HTL< MTL (Figure 1Aii). Meanwhile, these differences were not significant (*p* > 0.05) and had a positive correlation (R^2^ = 0.12) with δ15N values (Figure S2Bii), showing the possible biomagnification. The mean concentrations of the other elements were 8457.1, 8052.8, and 7296.3 mg/kg for LTL, MTL, and HTL, respectively, in the muscles of CE aquatic organisms, showing the following magnitude order: HTL < MTL < LTL (Figure 1A). Meanwhile, there are no significant differences between TLs (*p* > 0.05), and there is a negative correlation (R^2^ = 0.02) with δ15N values (Figure S2Biii), indicating that these elements are subjected to biodilution. This may be because the aquatic organisms in HTL are mainly carnivores, and most of them maintain a comparatively large body and actively prey on other animals that need higher nutrients for active behaviors. They might use the Other and EBTEs actively and more effectively than NETs, which are bioaccumulative. 

The findings of this study on accumulations of elements at the TL were consistent with previous studies. Madgett et al. [8] examined Hg, Cu, Cd, Ni, and Zn in the muscles of some aquatic species collected from Scotland. They found that there was a significant relationship with TLs, and benthic invertebrates showed species-specific accumulation of Cu, Cd, Ni, and Zn due to biomagnification. Some studies have reported bioaccumulation of non-essential hazardous heavy metals in fish tissues [7,54]. The analysis of elements (Cu, Pb, As, Se, Zn, Cr, Fe, Mn, Cd, and Hg) in the muscles of aquatic organisms, including *L. nebulosus* and *Epinephelus* spp., at the Red Sea [7] showed that all the elements were much higher than the recorded value in this study, except Zn, which showed a marked reduction. The concentrations of some elements (Cr, Mn, Co, Ni, Cd, and Pb) examined in *S. indicus* and *P. monodon* collected in Visakhapatnam, India [55] are much higher than our findings, except Fe and As in *P. monodon* and Zn in *S. indicus*; meanwhile, Cu was comparable to our findings. These results indicated that the concentration of different elements shows different accumulation mechanisms among the same species in different locations, whereas the concentrations of elements between different species are dissimilar. 

### 3.2. Levels of Toxic and Other Elements in Aquatic Organisms

Considering EBTEs and NETs in OP organisms, *Mobula kuhlii*, *Loligo duvauceli*, *Katsuwonus pelamis*, and *Risoprionodon acutus* comparatively showed elevated levels (Figure 2Ai,ii). For the CE organisms, the EBTE and NET content were higher in *Penaeus monodon*, *Lethrinus nebulosus*, *Lutjanus fulviflamma*, *Areus caelatus*, and *Lutjanus rivulatus* than other organisms (Figure 2Bi,ii). Our previous study on HPAHs also revealed that some of these species (*R. acutus*, *L. duvauceli*, *L. nebulosus*, and *A. caelatus*) showed higher levels than other studied organisms [14]. The organisms whose muscle contained comparatively higher levels of bioaccumulative elements include some planktivorous large ray (e.g., small fish and crustaceans eating cephalopods) and piscivorous echinoderm, and mollusks eating fishes included in different TLs (Table S1). These findings suggest that apart from TL transfer of heavy metals, there could be other infrequent opportunistic sources, including ingestion of the food item, which could be contaminated with the certain elements from the environment where accidental inputs have occurred. 

This study used the lowest MAL given to an element referring to the available literature (Table S4). Nine species studied exceeded the MAL at least for a single element. The EBTEs, such as Sn, Fe, Cu, and Zn, were below the MALs (Tables S2 and S3) for all the OP organisms, whereas the Cr levels exceeded the MAL (0.1 mg/kg) which is the Brazilian limit [56] for *R. kanagurta*, *M. kuhlii*, and *A. thazard*. There was no MAL for Cr set either by European Community (EC) regulations [57] or [58]; however, Copat et al. [57] has been used in the US EPA RfD to estimate Cr levels using Cr (III) (1.5 μg/g ww) and Cr (IV) (3 μg/g ww) [57,59]. Our study considered the MAL of Cr was 0.1 mg/kg ww according to the available permissible limit of Brazilian regulatory limit [56,58]. Additionally, there was no previous evidence for estimating the MAL of Cr using the wet weight of fish tissue, though some studies discussed its concentration given in dry weights (e.g., [60,61]). Therefore, the data obtained in this study cannot be compared. The concentrations of NETs, such as Sb and Hg, were below the MAL of all OP organisms. However, the concentration of As exceeded the MAL (3 mg/kg) [62] of *M. kuhlii*, *K. pelamis* (1.5 kg), and *R. acutus* and, Cd (MAL: 0.05 mg/kg) [63] for *L. duvauceli*, and Pb (MAL: 0.2 mg/kg) [63] for *S. albella* (Table S3). None of the elements of EBTEs detected in CE organisms exceeded the MALs. However, only As exceeded the MAL of NETs in *L. nebulosus* and *L. rivulatus* in the CE organisms, while other elements were within the given limits (Tables S2 and S3). 

**Figure 3:**
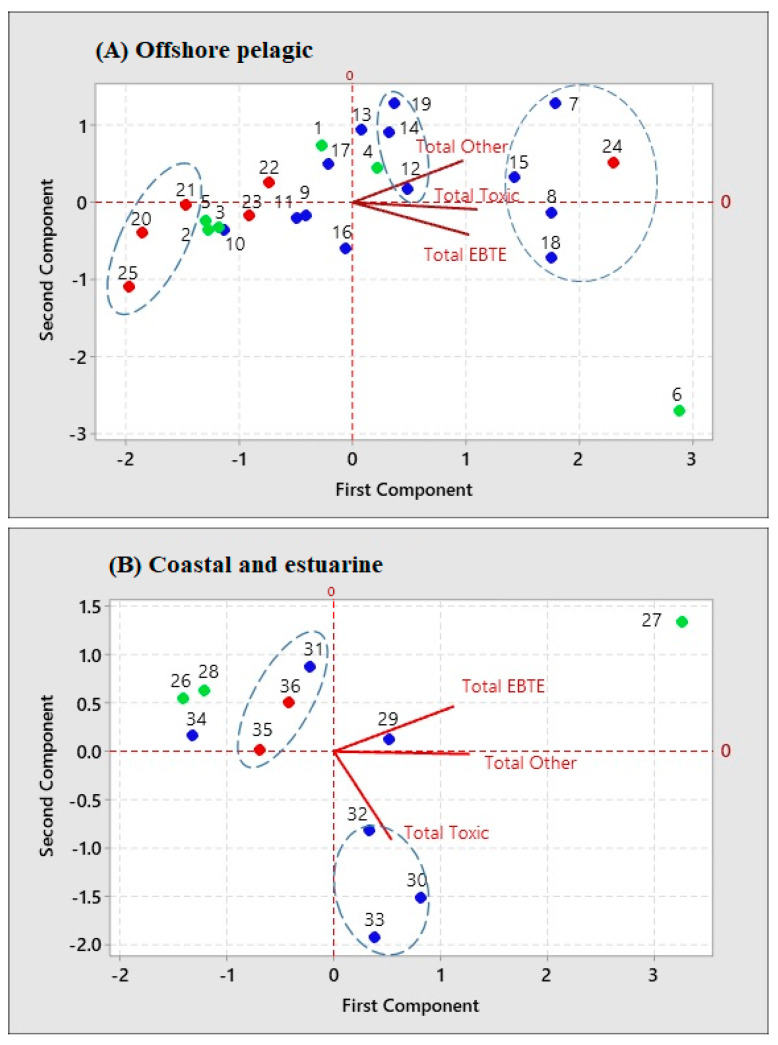
PCA biplot of elements (total EBTEs: essential but toxic in an excess amount, total NETs: non-essential toxic, and total Other) in muscle samples of offshore pelagic (**A**) and coastal and estuarine (**B**) organisms caught in Sri Lanka. Green: lower, blue: middle, and red: higher trophic level. 1: *S. commersonii*, 2: *G. minuta*, 3: *A. clupeoides*, 4: *R. kanagurta*, 5: *S. crumenophthalmus*, 6: *M. kuhlii*, 7: *L. duvauceli*, (8, 15, 18): *K. pelamis*, 9: *A. thazard*, 10: *D. russelli*, 11: *S. albella*, (12, 14, 16, 17, 19, 23): *T. albacares*, 13: *S. indicus*, 20: *A. solandri*, 21: *C. sexfasciatus*, 22: *I. platypterus*, 24: *R. acutus*, 25: *T. audax*, 26: *S. javus*, 27: *P. monodon*, 28: *O. niloticus*, 29: *C. caerulaurea*, 30: *L. nebulosus*, 31: *A. caelatus*, 32: *L. fulviflamma*, 33: *L. rivulatus*, 34: *S. ghobban*, 35: *S. sihama, and* 36: *E. malabaricus* (Table S1).

The principal component analysis (PCA) is an effective tool for identifying the homogeneous group of individual aquatic organisms. It is used to distinguish several possible groups accumulating each element category. The PCA of the total concentrations of element categories in the OP organisms resulted in the first two components showing 83%-variance and component 1 responsible for 59.7%-variance. Component 1 in the PCA derived to OP was responsible for a large positive correlation with total EBTEs, NETs, and other elements (eigenvectors were 0.57, 0. 61, and 0.54, respectively). In contrast, Component 2 had a negative correlation with total EBTEs and NETs, and a large positive correlation with total other elements (eigenvectors were −0.61, −0.13, and 0.79, respectively). The ID numbers 15, 8, and 18 were *K. pelamis,* while 12, 14, and 19 were relatively body-sized sized *T. alb*acares (Table S1), and 7 was a squid (*L. duvauceli*) belonging to MTL, together with the ID number 24, which was a shark (*R. acutus*) in HTL (Figure 3A), possibly making one cluster those whose diets mostly include small fishes and crustaceans (Table S1). The first principal component accounts for most of the variance, and the first eigenvector has all positive coordinates, meaning that all variables were positively correlated with each other. The second principal component had a much smaller variance than the first component. The scores of the PCA in the OP were well clustered; thus, they respond to the system quite uniformly, except some outsiders, such as *M. kuhlii* (ID: 6). A large manta ray, cartilaginous fish, and planktivorous feeding on small fish showed an isolated grouping from others. Moreover, the ID numbers 20, 21, and 25 represent large predatory fishes, such as *A. solandri*, *C. sexfasciatus*, and *T. audax,* respectively, in the HTL (Figure 3A) group. 

In the PCA of the total concentrations of element categories in CE organisms, the first two components explained 93.5% of the variation in the data in which the first component was responsible for 59.2%-variance. The CE organisms had a positive correlation with the concentration of the total EBTEs, NETs, and other elements (eigenvectors were 0.63, 0.30, and 0.71, respectively) in component 1 and a negative correlation with the total NETs and other elements in component 2 (eigenvectors: -0.89 and -0.02) and for the total EBTEs, it was 0.46. The eigenvector in the first component had all positive coordinates, implying that all variables were positively correlated with each other. The ID numbers 31 (*A. caelatus*) 35 (*S. sihama*) belonging to HTL, and 36 (*E. malabaricus*) belonging to MTL are predatory fishes mostly feeding invertebrates and small fishes in the CE, grouped in one cluster, while the *P. monodon* (ID: 27), which is a shrimp in the LTL feeding on detritus, benthic amphipods, and polychaetes was separated from the grouping (Figure 3B). The ID numbers 30 (*L. nebulosus*), 32 (*L. fulviflamma*), and 33 (*L. rivulatus*) belong to MTL and primarily feed on crustaceans, mollusks, and small fishes. Furthermore, the ID number 26 (*S. javus*) feeds on encrusting algae, 28 (*O. niloticus*) is an omnivore that feeds primarily on algae and plant matter belong to LTL, and 34 (*S. ghobban*) feeds on benthic algae and corals belong to MTL made a separate group (Figure 3B). Some organisms belong to different TLs but showed almost similar feeding habits and were grouped into the same cluster in PCA. The reason may be the species-specific preference for a particular food item to show different δ^13^C and δ^15^N ratios in their foods; therefore, the concentrations of elements may be different in the prey species. 

### 3.3. Toxic Element Concentrations in Yellowfin and Skipjack Tuna Fish

**Figure 4:**
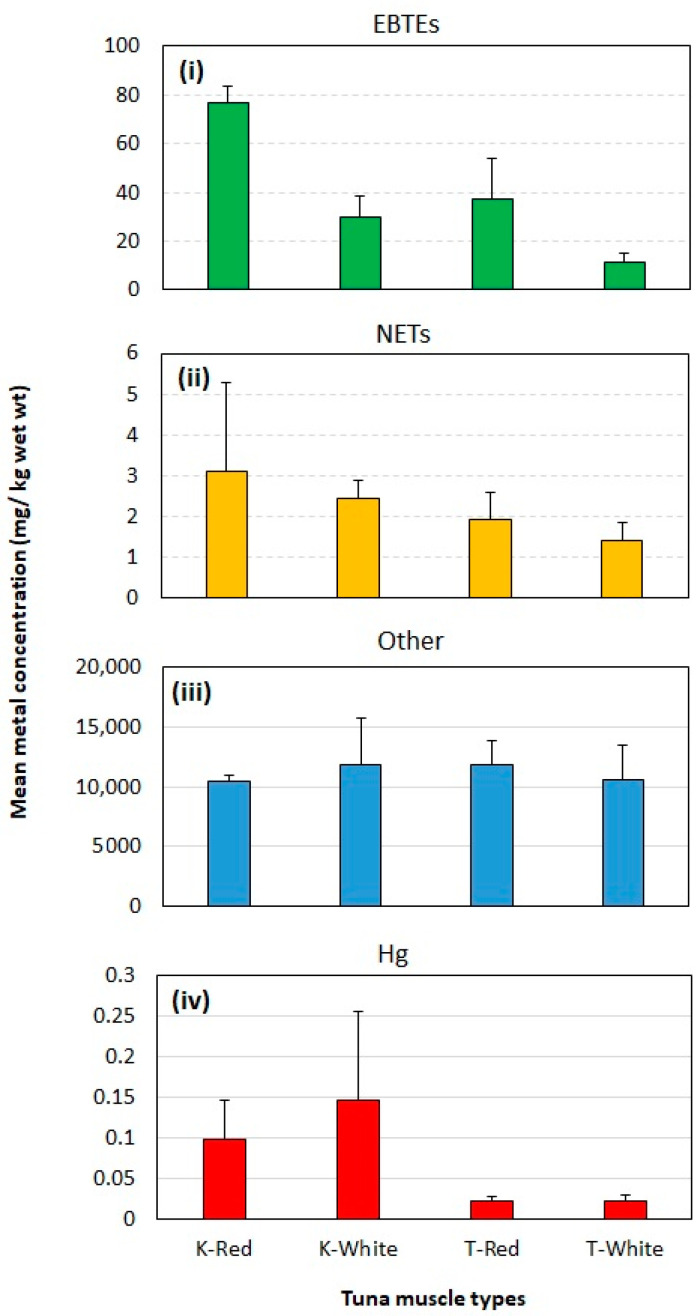
Mean concentration + standard deviation (SD) (mg/kg ww) of the three element groups (EBTEs: essential but toxic in excess quantities (**i**), NETs: non-essential toxic (**ii**), and Other (**iii**)), and mercury (**iv**) in the white and red muscles of yellowfin tuna (T: *T. albacares*), and skipjack tuna (K: *K. pelamis*) collected from Sri Lanka.

YFT and SJT belonging to the tuna fish category were the most dominant and frequently found in fish catch in Sri Lanka [64], especially focused on these species to evaluate EBTEs and NETs. There was a significantly different EBTEs content between fish and muscle types, but the NETs and other element contents did not significantly vary with fish or muscle types. The content of EBTEs between YFT and SJT showed significant differences (Two-way ANOVA: MS = 2494.4, F = 23.68, DF = 1, *p* = 0.001,). They also showed significant differences between red and white muscles (Two-way ANOVA: MS = 3933.5, F = 37.34, DF = 1, *p* = 0.0001) (Figure 4i). The EBTE content in the red muscles (76.6 ± 6.77) of SJT showed more than two and half the content of white muscles (29.8 ± 8.75) (Figure 4i). Similarly, the red muscles (37.1 ± 16.6) of YFT contained more than three times higher EBTEs than that of white muscles (11.5 ± 3.65) (Figure 4). The same trend was depicted by the NETs, where there were comparatively higher concentrations of elements (mg/kg) in the red muscle (SJT: 3.1 ± 2.18; YFT: 1.93 ± 0.65) than the white muscles (SJT: 2.45 ± 0.45; YFT: 1.4 ± 0.45) in both tuna species (Figure 4ii). The mean concentration of the other elements in the red muscles of YFT was 11809.7 ± 2122.6, which was comparatively higher than those element contents in the white muscles (10549.3 ± 2978.4), and the differences were not significant (*p* > 0.05). However, the other elements in the red muscle in SJT (10426.3 ± 556.0) showed marked reduction compared to its white muscle (11911.9 ± 3836), and the differences were not significant (*p* < 0.05) (Figure 4iii). Different fish species accumulate elements at different levels and to different rates, while different muscle types, such as white and red muscles, absorb nutrients and pollutants differentially because they have different functions [10].

Bosch et al. (2016) [29] revealed that the red muscles of YFT accumulate more total and inorganic Hg than the white muscles. Vieira et al. (2017) [65] also examined that the red muscles of SJT had a comparatively higher concentration of Hg than the white muscle. In contrast, Karunarathna and Attygalle [66] reported a relatively higher content of Hg in white muscles than in red muscles of YFT and SJT. This study reported nearly equal content of Hg between red and white muscles of YFT, but a higher level of Hg in the white muscles of SJT than in red muscles (Figure 4iv). Overall, white muscles of YFT and SJT contained comparatively lower levels of EBTEs (Figure 4i) and NETs, including Hg (Figure 4ii), than in red muscle. 

### 3.4. Mercury Accumulation in Yellowfin Tuna

The concentration of Hg previously did not show a positive correlation with the bodyweight of YFT [67]; however, others revealed a positive correlation [24]. This study found a significant relationship between the bodyweight of YFT (*n* = 7) and the concentration of Hg (ANOVA: MS = 0.126, F = 29.97. DF = 1, R^2^ = 85.7, *p* = 0.003), suggesting a biomagnification in the tissues (Figure S3). The mean Hg level in YFT of 0.16 ± 0.16 mg/kg ww (ranging from 0.02 to 0.43 mg/kg ww) in this study was lower than the previously reported values in Sri Lanka by Jinadasa et al. [24] (Table 1). This could be because the Hg level could be associated with the capture locations of fish [68]. Thus, the YFT is probably associated with areas that may be polluted with higher levels of Hg and ingested foods with elevated concentrations of Hg. Moreover, the recorded mean Hg levels in YFT in the Pacific and Atlantic Oceans were higher than the Hg level recorded in this study (Table 1). According to fishery management perspectives on sustainable utilization of ocean resources, and the quality and safety of seafood consumption, this study highlights that it is worthwhile to utilize larger and matured tuna fish that reproduced at least one time and helped to maintain tuna stocks in the oceans as there are consumer preferences and huge demand on big-sized tuna. However, the flesh amount must be determined by setting proper dietary guidelines according to the amount of heavy metals and other bioaccumulative elements in that flesh.

### 3.5. Human Health Risk Assessment of Consuming Yellowfin Tuna

**Figure 5:**
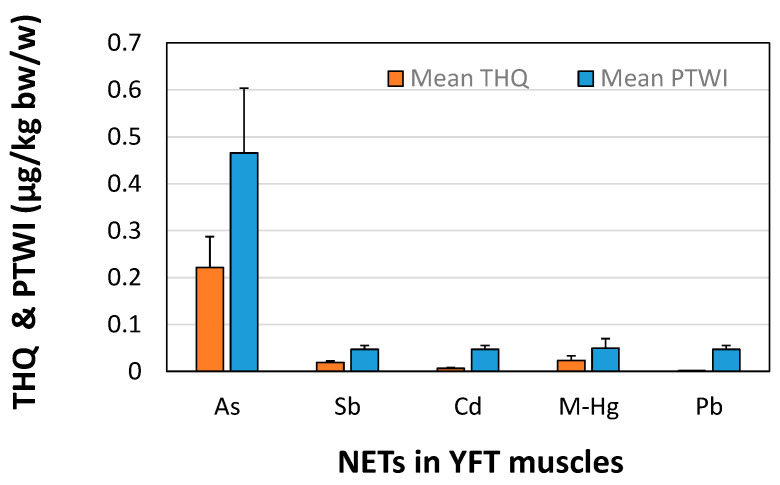
Target hazard quotient (THQ) and provisional tolerable weekly intake (PTWI) μg/kg bw/w of consuming yellowfin tuna (YFT, *T. albacares*). NETs: Non-essential toxic elements.

The human risk assessments for all NETs detected in YFT, a popular fish eaten by Sri Lankans, were evaluated using several replicate analyses. The estimated THQs for As, Sb, Cd, Hg, and Pb were 0.22 ± 0.07, 0.019 ± 0.003, 0.0067 ± 0.001, 0.024 ± 0.001, and 0.0016 ± 0.0003, respectively (Figure 5). The calculated HI for the non-carcinogenic health was 0.27, less than 1, suggesting that the lifetime exposure to these elements may not result in chronic risk posing adverse health effects in Sri Lankan adults. Moreover, the EDI of NETs for Sri Lankan adults was 9.38 × 10^−5^ ± 1.58 × 10^−5^ mg/kg bw/day. The estimated PTWI (μg/kg bw/w) was 0.47 ± 0.13 for As, 0.05 ± 0.008 for Sb, Cd, and Pb, and 0.05 ± 0.02 for M-Hg (Figure 5). The HI and PTWI values were below the recommended limits, indicating that the consumption of YFT is safe for Sri Lankan adults.

A previous study on non-carcinogenic health risks for adult Sri Lankans that estimated THQs, HI, and PTWI for YFT of Cd and M-Hg [9] found that the THQ-Cd is seven times higher, and THQ-M-Hg is five times lower than their corresponding safe values, which were 0.001 and 0.1, respectively (Figure 5). Furthermore, the estimated HI considering only M-Hg and Cd was 0.102, which was lower than our estimation [9]. The reason for higher HI in this study was more NETs, including As, Sb, Cd, Hg, and Pb, were considered for the HI calculation. The PTWI (μg/kg bw/w) calculated for M-Hg in this study is nearly four times lower than the PTWI–M-Hg (0.19) and six times higher than the PTWI-Cd (0.008) for Sri Lankan adults reported by Jinadasa et al. [9]. 

As it is the highest contributor among elements to estimate THQs, it shows the following magnitude order: Pb < Cd < Sb < Hg < As (Figure 5). Since it is the highest contributor to total THQs examined in fresh and processed tuna (*T. albacares* and *T. alalunga*) in Galicia, Spain, these values for adults were As: 2.8 × 10^−2^, Cd: 2.4 × 10^−3^, Pb: 2.2 × 10^−4^ [45]. Moreover, Saleh and Busaadia [72] revealed that the consumption of the studied four species, including *T. albacares*, may pose arsenic-related health risks to the Yemen population, though the other elements (Zn, Cu, Cd, and Pb) were within the safety limit. 

NETs have negative health effects on humans. The inorganic form of As was the most toxic as it was stable and soluble and efficiently absorbed by the human body [10,53]. Additionally, the organic form of As, primarily found in fish and meat [73], was the main source of dietary intake in humans [74,75] and showed a rapid excretion, so it does not accumulate [10,53]. Up to 90% of As in the fish muscles was in the form of arsenobetaine, which is the non-toxic organic form [76]. The reported human poisoning of As includes muscle weaknesses, abdominal pain, diarrhea, vomiting, skin flushing, and chronic exposure results in skin defects and cancer [53]. Sb is a chalcophilic group V metalloid and shows similar toxicity and geochemical behaviors as As [77,78]. Cd in fish muscles binds with proteins, so it is bioaccumulative; Cd can enter the fish body through gills via passive diffusion and from the food chain [17]. It is highly toxic to humans, causing carcinogenic effects, neurological disorders, hypertension, cardiovascular function, skeletal weaknesses, and defects [79]. Fish is easily absorbed Pb and accumulates in tissues, gills, bones, liver, and scales; thus, passing them to humans through the diet [80]. The organic Pb is more toxic than inorganic form [81]. Pb poisoning includes hematological effects, hypertension, neurological effects, renal failures, and cancer [81]. Human exposure to Hg is mainly due to the consumption of contaminated fish [50,82]. The organic form of Hg is considered to be more toxic and accumulates in fish tissue [83]. Inorganic forms of Hg are considered non-toxic; however, they can be converted to M-Hg, which is toxic [84]. The conversion can occur through a process catalyzed governed by bacteria in fish gills, gut, or photochemical reaction [83]. Once the M-Hg enters the human body, higher amount of them accumulates in the brain region [85], and the remaining parts accumulate in the liver, pituitary gland, and kidney [86]. Symptoms include headaches, movement difficulties, impaired vision and hearing, loss of coordination, tremors, ataxia, and paresthesia [87]. Therefore, evaluation of these NET elements in human food in temporal and spatial scales is very important.

## 4. Conclusions

This study investigated 36 elements, including toxic metals, where six elements were grouped as the EBTEs, five elements were grouped as the NETs, and the remaining twenty-five elements were grouped as Other in 26 of the commonly consuming aquatic organisms collected from OP and CE in Sri Lanka. The OP and CE dwelling organisms showed the same accumulation trend for the EBTEs between TLs, where the higher amount was detected at LTL (OP: 31.6; CE: 23.6) mg/kg, and a lower amount was found at HTL (OP: 11.4; CE: 8.82) mg/kg. In contrast, NETs were detected at higher levels at HTL for OP (1.89 mg/kg) and MTL for CE (1.99 mg/kg) organisms, indicating that essential elements are readily used by larger predators, while toxic elements are accumulating. The consumption of white muscles (EBTEs and NETs of YFT: [11.5 and 1.4] mg/kg and those for SJT were [29.8 and 2.45] mg/kg, respectively) of tuna appears to be safer than eating red muscles (EBTEs and NETs of YFT: [37.1 and 1.93] mg/kg and those for SJT were [76.6 and 3.1] mg/kg, respectively). Hg was accumulated when YFT grows; however, the concentrations were still below (0.16 ± 0.16 mg/kg ww) the regulation limit. Human health risk assessments on ingestion of NETs by consuming YFT showed that HI and PTWI values are below the recommended limits; thus, there was no potential human health risk for Sri Lankan adults by consuming YFT. The main advantage of this study was the analysis of EBTEs, NETs, and other elements in the commonly consuming aquatic organisms, considering their trophic levels after analyzing δ15N and δ13C signatures and considering MALs giving the special emphasis on the most popular YFT, and SJT. Moreover, another advantage was carrying out a toxicological risk assessment. Though the applied EDXRF technique was comparatively low cost and has a high performance, it also has the disadvantage of detecting elements in low concentrations. Therefore, we used NIC MA 3000 mercury analyzer to detect Hg in the samples. However, spatial and temporal scale monitoring of toxic elements in aquatic foods are compulsory since these elements could increasingly enter the aquatic environments due to anthropogenic and natural origins.

## Figures and Tables

**Table 1:** Comparisons of the inorganic mercury concentrations in yellowfin tuna (*T. albacares*).

Mean Hg Concentration (mg/kg)	Range	Mean Length (cm)/Weight (kg)	Method	Country	Ocean	Reference
0.16 ± 0.16 ^a^	0.02–0.43	118/*27*	Mercury analyzer (MA 3000, USA)	Sri Lanka	Indian Ocean	This study
0.30 ± 0.18 ^a^	(0.021) to 0.98 mg/kg	123.4/45.3	Cold vapor system atomic absorption spectrophotometry	Sri Lanka	Indian Ocean	[24]
0.26 ± 0.29 ^c^			Atomic absorption spectrophotometry	Sri Lanka	Indian Ocean	[19]
0.51 ± 0.33 ^b^	-	74.3 ± 11.4	Atomic absorption spectrophotometry	Baja California Sur, California	Eastern Pacific Ocean	[69]
0.98 ± 0.69	-	92.2 ± 19.5	Atomic absorption spectrophotometry	Equatorial Zone	Eastern Pacific Ocean	[70]
0.51 ± 0.32	-	22 kg/109 cm	Advanced mercury analyzer (combustion analyzer ALTEC 254)	Mozambique channel	Western Indian Ocean	[71]
0.70 ± 0.49	-	24 kg/104 cm	Advanced mercury analyzer (combustion analyzer ALTEC 254)	Reunion Island	Western Indian Ocean	[71]
0.77 ^a^	0.45 to 1.52 mg/kg wet weight	29.0 to 50.8 kg	Inductively coupled plasma mass spectrometry	South Africa	South Atlantic	[29]
2.75 ± 0.98 ^b,d^	1.51–4.49 ng/g dry wt	138.8 cm forked length/58.7 kg	Cold vapor atomic absorption spectroscopy	West Africa	north Atlantic Ocean	[28]
159 ± 79	48 to 500 ng·g^−1^ wet weight	92 ± 28 cm (forked length/16.8 ± 13.3 kg	Cold vapor atomic absorption spectrometry	Brazil	Equatorial Atlantic Ocean	[67]

^a^: wet weight; ^b^: dry weight; ^c^: cooked; ^d^: ng/g.

## Data Availability

The data presented in this study are available on request from the corresponding authors.

## Supplementary Materials

The following supporting information can be downloaded at: https://www.mdpi.com/article/10.3390/toxics10100585/s1, Figure S1: Relationships between δ13C and δ15N (‰) in aquatic species collected from offshore pelagic (**A**) and coastal and estuarine ecosystems (**B**) in Sri Lanka; Figure S2: δ15 N verses concentrations of EBTEs, NETs and Other elements between off-shore pelagic (**A**) and coastal and estuarine (**B**) aquatic organisms collected in Sri Lanka; Figure S3: Relationship between the concentration of mercury (mg/kg ww) and body weight of yellowfin tuna (*T. albacares*) collected in Sri Lanka; Table S1: Details of aquatic species and their information; Table S2: Concentration of toxic and other elements (mg/kg ww) in off-shore pelagic edible aquatic organisms collected in Sri Lanka; Table S3: Concentration of toxic and other elements (mg/kg ww) in coastal and estuarine edible aquatic organisms collected in Sri Lanka; Table S4: The maximum allowable limits of toxic elements and the regulatory bodies [88,89,90,91,92,93].
