# Supplementary material for: Comparative Study on the Distribution of Essential, Non-Essential Toxic, and Other Elements across Trophic Levels in Various Edible Aquatic Organisms in Sri Lanka and Dietary Human Risk Assessment
Source: Toxics. 2022 Oct 4;10(10):585. doi: 10.3390/toxics10100585 (PMC9612099; doi:10.3390/toxics10100585)
Supplement: Supplementary file 1 [file toxics-10-00585-s001.zip › toxics-1932966-supplementary.pdf]

toxics MDPI

Supplementary Materials: Comparative Study on the Distribution of Essential, Non-essential Toxic, and Other Elements Across Trophic Levels in Various Edible Aquatic Organisms in Sri Lanka and Dietary Human Risk Assessment

Anura Upasanta-Kumara Wickrama-Arachchige, Keerthi S. Guruge, Hinako Tani, Tilak Siri Dharmaratne, Marappullige P. Kumara, Yasuaki Niizuma and Takeshi Ohura

Table S1. Details of aquatic species and their information.

#	Scientific Name	English Name	n	Habitat Type	Feeding Type	Maximum Length Recorded / (Length Recorded in This Study) (cm)	Weight (g)
**Off-shore pelagic species in Sri Lanka (OPSL)**							
1	*Stolephorus commersonii*	Commerson's anchovy	14	Mud and sand bottoms.	Benthic, feeding at bottom at daytime but leave the banks at sunset.	10/(8)	7
2	*Gazza minuta*	Toothpony	10	Shallow coastal waters, close to bottom.	Small fishes, Polychaetes and crustaceans.	14/(9)	8
3	*Amblygaster clupeoides*	Bleeker's smoothbelly sardinella	5	Pelagic, enter shallow waters frequently.	Plankton feeders.	28 usually 20/ (16)	35
4	*Rastrelliger kanagurta*	Indian mackeral	3	Pelagic, neritic.	Mainly on zooplankton, primarily crustaceans.	35/(21)	180
5	*Selar crumenophthalmus*	Bigeye scad	3	Semi-dermasal, neritic, mud and sand, sandstone and coral, enter harbours, come close to shore.	Feed close to bottom at daytime, planktonic feeder, small benthic invertebrates.	35/(13)	40
6	*Mobula kuhlii*	Lesser devil ray	1	Pelagic, often coming close inshore near coral reefs.	Planktivore, small fish.	670 (disc width)	56400
7	*Loligo duvauceli*	Indian squid	3	Neritic, shallow water depths of 30 m to 170 m.	Small fish, crabs, and shrimp.	29/(27) (from tip of the tentacle to posterior fin)	100
8, 15, 18	*Katsuwonus pelamis*	Skipjack tuna	3	Pelagic, oceanic, restricted to continental slopes.	Small fishes, cephalapods and crustaceans.	105/(43) (44) (45)	1400 1450 1500
9	*Auxis thazard*	Frigate tuna	1	Pelagic.	Small pelagic fishes, planktonic crabs, shrimps and stomatopod larvae.	60/(37)	950
10	*Decapterus russelli*	Indian scad	3	Pelagic, mid water or surface, over deep waters 20 to 100 m, enter deep	Smaller planktonic invertebrates.	40/(30)	140

				harbours seasonally, move to surface at night.			
11	*Sardinella albella*	White sardinella	10	Pelagic, enter shallow waters frequently, rock or coral reefs.	Plankton feeders.	14/(14)	20
12,<br>14,<br>16,<br>17<br>19,<br>23	*Thunnus albacares*	Yellowfin tuna	7	Pelagic oceanic, but come to shallow waters, close to land relatively shallow waters migratory, above and below thermocline.	Pelagic crustaceans, small fishes and squid.	200/ (36)<br>(35)<br>(159)<br>(161)<br>(35)<br>(103)<br>(163)	1200<br>1400<br>71000<br>73000<br>1400<br>27000<br>75000
13	*Stolephorus indicus*	Indian anchovy	10	Occasional on mud and sand.	Benthic, feeding at bottom at daytime but leave the banks at sunset.	16/(14)	18
20	*Acanthocybium solandri*	Wahoo	1	Pelagic, generally offshore, but comes close to shore over deep clear water.	Pelagic fishes and squids.	215	15000
21	*Caranx sexfasciatus*	Bigeye trevally	3	Semi demersal close to bottom, sand stone and coral depth to 50 m, juveniles in estuaries.	Crustaceans, squids and cuttlefish and small fish.	90/(16.5)	55
22	*Istiophorus platypterus*	Indo-pacific sailfish	1	Epipelagic, generally beyond continental shelf, generally above the thermocline, Migratory.	Feed on large fishes, cephalopods.	340	80000
24	*Risoprionodon acutus*	Milk shark	1	Inshore and Offshore surface to depths of 50 m.	Small bony fishes and crustaceans.	102	8000
25	*Tetrapturus audax*	Striped marlin	1	Epipelagic, highly migratory, Oceanic, usually swimming above the thermocline.	Feed on large fishes, cephalopods.	350	70000
Coastal and estuarine ecosystem species (CE)							
26	*Siganus javus*	Rabbit fish	3	Demersal, neritic, living in shallow coral and sandstone reefs, Juveniles in estuaries.	Feed on encrusting algae.	45/(16.5)	70
27	*Penaeus monodon*	Giant tiger shrimp	14	Adult in sea, juveniles in estuaries low salinity lagoons, quite abundant at mud and mixture of mud and sand down to depth 20 m in the sea.	Feed on detritus, Benthic amphipods, polychaetes.	Male is 27, female is 35/(17)	14.6
28	*Oreochromis niloticus*	Mozambique tilapia	3	Estuaries, lagoons and fresh water.	*Omnivore fish, feed* on algae, plant matter, organic particles, small invertebrates and other fish.	38/(19.5)	110
29	*Caesio caerulaurea*	Blue-and-gold fusilier	3	Coral and sandstone to a depth 30 m, large schools near the bottom.	Plankton feeders.	25/(20)	100
30	*Lethrinus nebulosus*	Head dot	3	Dermasal abundant to depth on 120 m, adult in coral and sandstone, Juveniles up to 6 cm in seagrass beds.	Adult stage feed on mollusks, crustaceans and echinoderms.	80/(23.5)	210
31	*Arius caelatus*	Engraved catfish	3	Marine and high salinity estuaries.	Benthic invertebrates mostly including Crustaceans such as crab and shrimps.	45/(30)	140

32	*Lutjanus fulviflamma*	Blackspot snapper	1	Inhabiting 10-60 m, usually in coral reefs and deep lagoons, juveniles sometimes found in mangrove estuaries or in the lower reaches of fresh water streams.	Predatory, feed crustaceans, bivalves, gstropods, cephalopods, and small fishes.	35/(26)	280
33	*Lutjanus rivulatus*	Blubberlip snapper	1	Hard bottom, living in shallow coral and sandstone reefs to depths 150 m, most abundant at 20 – 80 m.	Predatory, feed crustaceans, bivalves, gstropods, cephalopods, and small fishes.	75/(23.5)	570
34	*Scarus ghobban*	Yellow scale Parrot fish	1	On coral reefs, schools of 15-20 individuals.	Feed on benthic algae, corals.	60/(30.5)	470
35	*Silago sihama*	Silver sillago	1	Benthic, neritic, shallow sandy bottom of shores and bays and high salinity lagoons.	Crustacean, teleosts, mollusks, echinoderms and polychaetes feeder.	25/(19)	105
36	*Ephinephelus malabaricus*	Malabar grouper	1	Shallow (coral reefs and sandstone reefs) to 60 m depth, stay at mud banks at night.	Feed on small fishes.	100/(30.5)	280

#: Identification number of aquatic organism. The number is assigned to each of the organisms according to the trophic position of the stable isotope analysis Graph S1; n: number of individual organisms used to prepare one samples; Weight (g) is given to one organism; N/R=Not recorded; The habitat, feeding type and the length (cm) is given as the previously recorded maximum length of the particular species of Sri Lankan samples (DeBruin et al., 1994), and /(maximum length recorded in this study). The body weight is given where applicable for Sri Lankan samples as maximum body weight (g) of the species used in this study. Detail of the Japanese fish were taken from Fish base, 2019.

Table S2. Concentration of toxic and other elements (mg/kg ww) in off-shore pelagic edible aquatic organisms collected in Sri Lanka.

ID	Lower Trophic Level								Middle Trophic Level											Higher Trophic Level					
	**1**	**2**	**3**	**4**	**5**	**6**	**7**	**8**	**9**	**10**	**11**	**12**	**13**	**14**	**15**	**16**	**17**	**18**	**19**	**20**	**21**	**22**	**23**	**24**	**25**
**Essential but Toxic in Excess Amount (EBTEs)**																									
**Sn**	2.53	1.29	1.86	3.79	1.59	2.31	0.27	3.73	3.13	1.60	1.93	2.63	2.90	2.11	2.93	2.64	3.17	3.22	2.06	1.69	1.42	1.70	3.28	4.09	1.42
**Fe**	5.95	7.00	8.55	14.0	6.10	85.2	4.29	44.2	16.6	5.59	11.1	24.1	5.81	12.3	34.4	28.8	7.12	32.4	10.3	1.97	2.73	4.59	7.03	6.13	3.74
**Cu**	0.72	0.43	0.78	1.46	0.93	0.71	6.17	2.86	1.12	0.67	0.74	2.25	0.73	1.10	1.98	0.66	0.79	2.63	0.97	ND	0.47	0.51	0.70	ND	0.39
**Cr**	ND	ND	ND	0.29	ND	0.18	ND	ND	0.22	ND	ND	ND	ND	ND	ND	ND	ND	ND	ND	ND	ND	ND	ND	BDL	ND
**Zn**	10.2	5.00	5.90	7.28	4.77	8.18	18.8	8.51	6.71	5.32	8.32	4.75	9.92	3.49	7.44	4.18	4.75	7.68	3.53	3.23	2.97	7.99	3.98	4.67	3.09
**Se**	0.36	0.14	0.47	0.58	0.27	0.36	0.80	1.64	0.67	0.53	0.45	1.23	0.48	1.03	2.08	2.20	0.79	3.45	0.87	0.28	0.12	0.51	0.70	0.58	0.39
**Non-essential toxic (NETs)**																									
**As**	0.36	0.57	0.31	0.87	0.40	3.91	1.88	0.97	0.45	0.93	0.89	1.22	0.73	1.44	1.49	0.66	1.06	3.48	1.23	BDL	0.36	0.51	0.70	6.72	BDL
**Sb**	ND	ND	ND	ND	ND	ND	ND	0.53	ND	ND	ND	ND	ND	ND	ND	ND	ND	0.27	ND	ND	ND	ND	ND	ND	ND
**Cd**	ND	ND	ND	ND	ND	ND	1.07	ND	ND	ND	ND	ND	ND	ND	ND	ND	ND	ND	ND	ND	ND	ND	ND	ND	ND
**Hg***	ND	ND	ND	ND	ND	0.36	ND	ND	ND	ND	ND	ND	ND	ND	ND	0.22	ND	ND	ND	ND	ND	ND	ND	ND	0.64
**Hg****	0.003	0.002	0.006	0.001	0.005	0.31	0.02	0.21	0.07	0.01	0.03	0.02	0.07	0.02	0.06	0.22	0.27	0.06	0.02	0.02	0.01	0.1	0.15	0.39	0.48
**Pb**	ND	BDL	BDL	ND	BDL	0.18	ND	0.18	ND	ND	0.30	ND	BDL	BDL	ND	ND	ND	ND	BDL	ND	BDL	0.17	ND	ND	0.13
**Other elements**																									
**Mg**	397	123	263	425	227	244	375	491	266	226	238	391	470	314	408	292	377	366	290	167	205	270	279	374	93.2
**Al**	102	47.4	58.3	103	56.1	71.7	148	110	85.4	54.4	66.4	92.3	106	109	123	76.1	83.6	93.0	114	33.3	52.1	87.4	59.3	112	30.4
**Si**	64.2	31.4	33.7	65.0	31.0	30.1	58.5	46.4	35.3	20.2	49.0	36.9	44.1	31.0	40.8	34.7	41.9	37.5	31.0	15.6	21.7	28.7	38.9	45.3	14.8
**P**	1225	676	993	1760	912	1325	1738	2099	1172	869	999	1716	1469	1280	1637	1523	1791	1563	1182	891	891	1105	1305	2126	436
**S**	1598	1324	1467	2838	1604	1686	4720	3766	2073	1504	1678	2526	2662	1847	2832	1963	2414	2811	1681	985	1924	1870	2167	2158	485
**Cl**	4059	726	1218	1183	993	1063	4452	1212	1945	1344	1400	1866	2614	5113	4147	637	757	1237	6626	321	746	2414	419	1986	363
**K**	1434	1333	1663	3671	1643	2863	2387	4051	2281	1637	1120	3287	2086	2169	2801	3011	3746	3068	2022	2221	2078	2244	2332	4001	1080
**Ca**	1371	1400	402	186.75	553	58.5	226	172	69.8	74.1	1752	186	1639	321	284	67.5	78.6	85.1	279	27.7	207	77.0	59.3	93.8	39.1

**Ti**	ND	ND	ND	ND	ND	ND	ND	ND	ND	ND	0.59	ND	ND	ND	0.20	ND	ND	ND	0.31	ND	ND	ND	ND	ND	ND
**Mn**	ND	1.29	ND	ND	ND	0.89	ND	ND	ND	ND	1.93	ND	ND	ND	ND	ND	ND	ND	ND	ND	ND	ND	ND	ND	ND
**Co**	ND	ND	ND	ND	ND	ND	ND	ND	ND	BDL	ND	0.11	ND	ND	0.10	ND	ND	ND	BDL	ND	ND	ND	ND	ND	BDL
**Ni**	ND	0.29	ND	ND	ND	ND	ND	ND	ND	ND	ND	ND	ND	ND	ND	ND	ND	ND	ND	ND	ND	BDL	ND	ND	ND
**Ga**	ND	ND	ND	ND	ND	ND	ND	ND	ND	ND	ND	ND	ND	ND	ND	ND	ND	ND	ND	ND	ND	ND	ND	ND	ND
**Ge**	ND	ND	ND	ND	ND	ND	ND	ND	ND	ND	ND	ND	ND	ND	ND	ND	ND	ND	ND	ND	ND	ND	ND	ND	BDL
**Br**	5.41	2.57	2.49	4.95	3.05	4.80	13.68	5.24	3.35	2.00	2.52	3.74	7.26	3.49	4.89	3.08	2.90	4.59	3.95	1.12	2.14	8.33	2.11	8.47	4.38
**Rb**	0.36	0.43	0.31	0.58	0.27	1.07	1.07	0.72	0.45	0.27	0.30	0.54	0.48	0.34	0.48	0.44	0.53	0.56	0.50	0.42	0.36	0.51	0.47	0.88	0.26
**Sr**	3.43	14.28	0.78	1.46	3.45	0.36	1.88	0.97	0.45	0.27	4.90	0.56	7.26	1.87	1.93	BDL	BDL	0.42	1.65	ND	2.37	0.34	BDL	BDL	BDL
**Y**	ND	ND	ND	ND	ND	ND	ND	ND	ND	ND	ND	ND	ND	ND	ND	ND	ND	ND	ND	ND	ND	ND	ND	ND	ND
**Te**	ND	ND	ND	ND	ND	ND	ND	ND	ND	ND	ND	ND	0.97	0.31	ND	ND	ND	ND	0.32	0.42	ND	ND	ND	ND	ND
**I**	ND	ND	ND	ND	ND	ND	ND	ND	ND	ND	ND	ND	ND	ND	ND	ND	ND	ND	ND	ND	ND	ND	ND	ND	ND
**Hf**	0.72	0.71	0.78	0.87	ND	0.89	1.07	0.78	0.67	0.40	ND	0.66	0.97	0.68	0.86	0.66	1.06	0.84	0.84	0.70	0.47	0.68	0.47	1.75	0.39
**Ta**	0.18	BDL	0.31	0.58	BDL	0.36	0.80	BDL	0.22	BDL	0.15	0.46	0.24	0.06	0.10	ND	0.26	BDL	0.12	ND	ND	0.34	BDL	ND	BDL
**Pt**	BDL	BDL	BDL	BDL	ND	BDL	BDL	ND	BDL	ND	BDL	BDL	ND	ND	BDL	ND	ND	BDL	BDL	ND	ND	BDL	ND	ND	ND
**Au**	0.18	BDL	BDL	BDL	ND	BDL	BDL	BDL	BDL	BDL	BDL	BDL	0.24	ND	0.28	ND	ND	BDL	BDL	BDL	BDL	0.17	ND	ND	ND
**U**	ND	ND	ND	ND	ND	ND	ND	ND	ND	ND	ND	ND	ND	ND	ND	ND	ND	ND	ND	ND	ND	ND	ND	ND	ND

1: *S. commersonii*, 2: *G. minuta*, 3: *A. clupeoides*, 4: *R. kanagurta*, 5: *S. crumenophthalmus*, 6: *M. kuhlii*, 7: *L. duvauceli*, (8, 15, 18): *K. pelamis*, 9: *A. thazard*, 10: *D. russelli*, 11: *S. albella*, (12, 14, 16, 17, 19, 23): *T. albacares*, 13: *S. indicus*, 20: *A. solandri*, 21: *C. sexfasciatus*, 22: *I. platypterus*, 24: *R. acutus*, 25: *T. audax*.

Table S3. Concentration of toxic and other elements (mg/kg ww) in coastal and estuarine edible aquatic organisms collected in Sri Lanka.

ID	Lower Trophic Level			Middle Trophic Level						Higher Trophic Level	
	**26**	**27**	**28**	**29**	**30**	**31**	**32**	**33**	**34**	**35**	**36**
	**Essential but Toxic in Excess Amount (EBTEs)**										
**Sn**	1.07	4.10	1.51	2.59	2.76	1.48	2.09	2.57	1.70	1.63	1.99
**Fe**	2.03	25.9	2.01	5.89	2.30	8.27	4.38	3.30	2.08	1.88	2.17
**Cu**	0.36	10.7	0.38	0.71	0.92	0.37	0.83	0.55	0.38	0.50	0.54
**Cr**	ND	ND	ND	ND	ND	ND	ND	BDL	ND	ND	ND
**Zn**	2.15	17.04	3.27	4.95	3.91	16.41	4.17	4.04	3.59	4.27	4.51
**Se**	BDL	0.63	0.25	0.47	0.46	0.12	0.42	0.73	0.19	0.38	0.72
	**Non-essential toxic (NETs)**										
**As**	0.12	0.63	ND	0.71	2.30	0.49	1.88	3.30	0.57	0.88	BDL
**Sb**	ND	ND	ND	ND	0.46	ND	ND	ND	ND	ND	ND
**Cd**	ND	ND	ND	ND	ND	ND	ND	ND	ND	ND	ND
**Hg***	ND	ND	ND	ND	ND	ND	ND	ND	ND	ND	ND
**Hg****	0.002	0.009	0.002	0.006	0.09	0.17	0.13	0.11	0.002	0.06	0.09
**Pb**	BDL	ND	ND	BDL	BDL	ND	BDL	BDL	BDL	BDL	0.18
	**Other elements**										
**Mg**	178	691	193	427	420	80.3	344	206	26.8	172	258
**Al**	40.2	143	41.3	94.7	94.0	46.8	77.6	68.2	23.8	52.2	70.1
**Si**	15.0	160	23.6	39.4	37.9	15.6	31.9	37.4	9.82	19.6	26.4
**P**	541	2294	721	1838	1762	489	1397	947	395	721	1031
**S**	1359	2629	1319	3040	3101	1259	3086	2568	661	2247	2709
**Cl**	626	1575	484	1176	1008	1444	678	791	200	812	982
**K**	1323	2651	2072	4478	4525	1419	3503	3027	1964	2184	2962
**Ca**	132	5965	82.4	223	135	627	257	132	59.1	93.8	240
**Ti**	ND	2.52	ND	ND	ND	ND	ND	0.37	ND	ND	ND
**Mn**	ND	3.47	ND	ND	ND	ND	ND	ND	ND	ND	ND
**Co**	BDL	ND	BDL	ND	0.46	ND	BDL	ND	ND	ND	ND
**Ni**	ND	ND	ND	ND	ND	ND	ND	ND	ND	ND	ND
**Ga**	ND	ND	BDL	ND	ND	ND	ND	ND	ND	ND	ND
**Ge**	ND	ND	ND	ND	ND	ND	ND	ND	ND	ND	ND

**Br**	2.26	28.72	1.76	4.01	3.45	4.44	2.71	3.67	2.08	2.76	4.33
**Rb**	0.36	1.89	4.02	0.71	0.69	0.62	0.42	0.73	0.76	0.38	0.72
**Sr**	0.83	61.22	0.38	1.18	0.46	7.16	1.88	0.55	BDL	0.63	1.26
**Y**	ND	ND	BDL	ND	ND	ND	ND	ND	ND	ND	ND
**Te**	ND	ND	ND	ND	ND	ND	ND	ND	ND	ND	ND
**I**	ND	ND	ND	ND	ND	ND	ND	ND	1.51	ND	ND
**Hf**	0.36	0.95	0.25	0.94	ND	0.62	0.83	0.73	0.57	0.38	0.72
**Ta**	BDL	1.58	BDL	0.24	BDL	0.25	0.21	0.18	0.19	0.13	ND
**Pt**	ND	BDL	BDL	ND	BDL	BDL	BDL	ND	ND	ND	BDL
**Au**	ND	0.32	BDL	BDL	ND	0.25	ND	BDL	BDL	ND	BDL
**U**	ND	ND	BDL	ND	ND	ND	ND	ND	ND	ND	ND

ND: Not detected; BDL: Below detection limit; Hg*: XDRF detection; Hg**: MA 3000 detection; Both Hg* and Hg** not belong to M-Hg; 26: *S. javus*, 27: *P. monodon*, 28: *O. niloticus*, 29: *C. caerulaurea*, 30: *L. nebulosus*, 31: *A. caelatus*, 32: *L. fulviflamma*, 33: *L. rivulatus*, 34: *S. ghobban*, 35: *S. sihama*, and 36: *E. malabaricus*.

Table S4. The maximum allowable limits of toxic elements and the regulatory bodies.

**ID**	**MAL (mg/kg)**	**Specifications**	**Regulatory body**	**Reference**
		Essential but Toxic in Excess Amount (EBTEs)		
Sn	50	For all uncanned meat and meat production	South African Department of Health	[62]
Fe	100	Fresh fish muscles<br>There is no maximum permissible level has been set in regulations [83]	WHO	[88], [89]
Cu	30	Fresh weight of fish	FAO	[54], [90]
	20	Fresh weight of fish	UK, Spain	[54]
	5	Fresh weight of fish	Turkey	[54]
Cr	0.1	Fish food<br>(The<br>adequate dietary intake in adults can range from 0.50 to 2.00 lg (Cr (III)), WHO 1996)	Brazilian standards	[56]
Zn	1000	Fresh fish	National Health and Medical Research Council (NHMRC)	[91]
	30		FAO	[90]
Se	5 mg/kg *(Dry mass)*	Human foods	Australia New Zealand Food Authority	[60], [92]
	3 mg/kg (Dry wt)	Tissue concentration	U.S. Forest Service, with the National Environmental Policy Act	[61]
		Non-Essential Toxic (NETs)		
As	3.0	Fish and processed fish meat	South African Department of Health	[62]
	2.0	Fish and processed fish meat	Australia and New Zealand food authority, 2011	[54]

Sb	0.15	All liquid foodstuffs	South African Department of Health	[62]
Cd	0.05	Tuna (*Thunnus* species and *Katsuwonus pelamis*)	FAO, Heavy Metal Regulations Legal Notice No. 66/2003	[63]
	0.1	Tuna (*Thunnus* species and *Katsuwonus pelamis*)	Commission Regulation (EC) No. 1881/2006	[59]
	1.0	Fish and processed fish	South African Department of Health	[62]
	0.3	Anchovy, sword fish	Commission Regulation (EC) No. 1881/2006	[59]
Hg	1	Predatory fish including shark, sailfish, tuna, pike and other high-mercury fish	The United Kingdom and the European Union	[62]
	0.5	Edible parts of fishery products	South African Department of Health, WHO	[62]
M-Hg	1	Fresh fish muscles	The U.S. Food and Drug Administration (FDA) has an action level for methylmercury	[49], [93]
Pb	0.5	Fish and processed fish	South African Department of Health	[62]
	0.2	Edible parts of the fishery products	FAO, Heavy metal regulations legal notice no. 66/2003	[63]
	0.3	Muscle meat of fish	Commission regulation (EC) no. 1881/2006	[59]
	2	Fresh weight basis of fish	Australian National Health and Medical Research Council (ANHMRC)	[91]

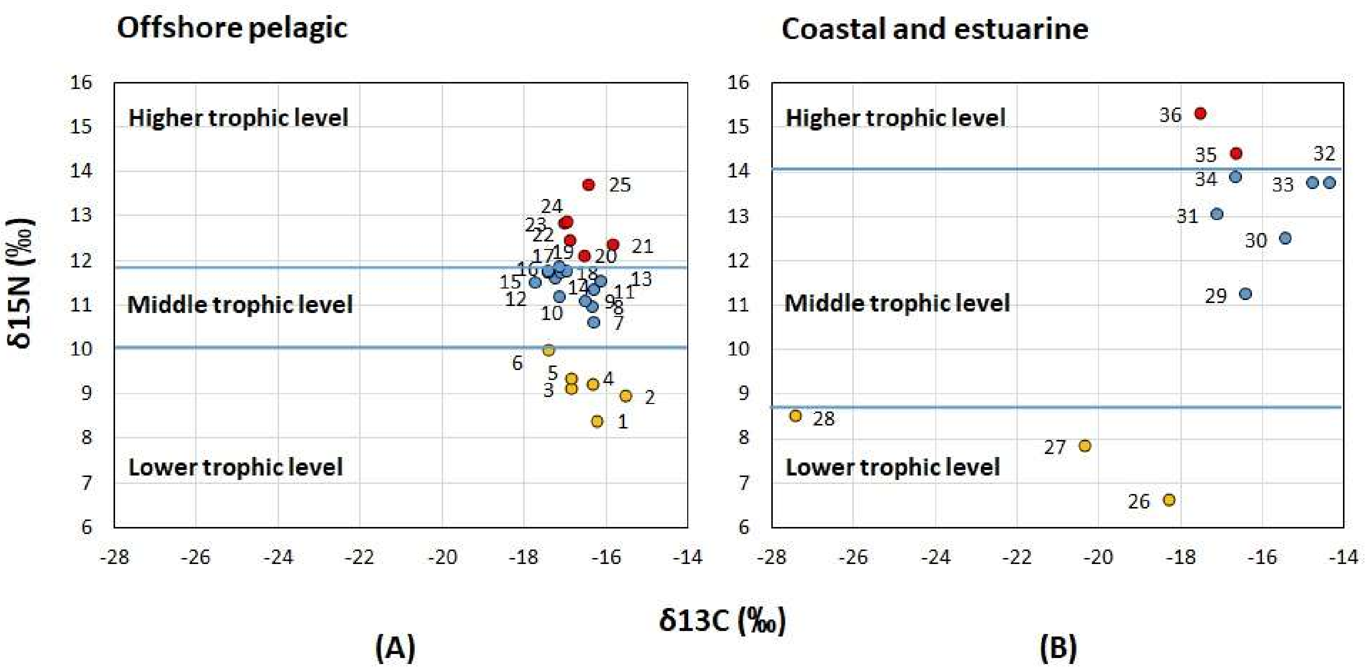

Figure S1. Relationships between δ13C and δ15N (‰) in aquatic species collected from offshore pelagic (**A**) and coastal and estuarine ecosystems (**B**) in Sri Lanka. The numbers correspond to those in Table S2. such as 1: *S. commersonii*, 2: *G. minuta*, 3: *A. clupeoides*, 4: *R. kanagurta*, 5: *S. crumenophthalmus*, 6: *M. kuhlii*, 7: *L. duvauceli*, (8, 15, 18): *K. pelamis*, 9: *A. thazard*, 10: *D. russelli*, 11: *S. albella*, (12, 14, 16, 17, 19, 23): *T. albacares*, 13: *S. indicus*, 20: *A. solandri*, 21: *C. sexfasciatus*, 22: *I. platypterus*, 24: *R. acutus*, 25: *T. audax*, 26: *S. javus*, 27: *P. monodon*, 28: *O. niloticus*, 29: *C. caerulaurea*, 30: *L. nebulosus*, 31: *A. caelatus*, 32: *L. fulviflamma*, 33: *L. rivulatus*, 34: *S. ghobban*, 35: *S. sihama*, and 36: *E. malabaricus*.

The lower trophic level of the food chain of the offshore pelagic ecosystem included *Stolephorus commersonii* (the species number in Figure 1, 1), *G. minuta* (2), *A. clupeoides* (3), *R. kanagurta* (4), *S. crumenophthalmus* (5), and *M. kuhlii* (6) where there were comparatively small fishes and a large planktivore ray were represented in which δ^{15}N ranged from 8.38 to 9.96‰ and δ^{13}C ranged from -15.5 to -17.4‰. The middle trophic level of the food chain of the above ecosystem included *L. duvauceli* (7), *K. pelamis* 1.4 kg weight (8), *A. thazard* (9), *D. russelli* (10), *S. albella* (11), *T. albacares* 1.2 kg weight (12), *S. indicus* (13), *T. albacares* 1.4 kg weight (2nd sample) (14), *K. pelamis* 1.45 kg weight (15), *T. albacares* 71kg weight (16), *T. albacares* 73kg weight (17), *K. pelamis* 1.5 kg weight (18), and *T. albacares* 1.4 kg weight (1nd sample) (19) and the assigned δ^{15}N ranged from 10.6 to 11.8‰ and δ^{13}C ranged from -16.1 to -17.7‰. Comparatively large predatory fishes such as *A. solandri* (20), *C. sexfasciatus* (21), *I. platypterus* (22), *T. albacares* 27kg weight (23), *R. acutus* (24), and *T. audax* (25) were classified to higher trophic level of the OP ecosystem in which the δ^{15}N ranged from 12.1 to 13.7‰ and δ^{13}C ranged from -15.8 to -17‰.

For CE ecosystem, the species *S. javus* (26), *P. monodon* (27), and *O. niloticus* (28), were classified as lower trophic level according to their corresponding δ^{15}N values (6.61 to 8.49‰) and δ^{13}C values (-18.3 to -27.4‰) and their general feeding habits. The species *C. caerulaurea* (29), *L. nebulosus* (30), *A. caelatus* (31), *L. fulviflamma* (32), *L. rivulatus* (33), and *S. ghobban* (34), were grouped in the middle trophic level of CE ecosystem in which the δ^{15}N ranged from 11.2 to 13.9‰, and δ^{13}C values ranged from -14.3 to -17.1‰ whereas *S. sihama* (35) and *E. malabaricus* (36) (δ^{15}N: from 14.4 to 15.3‰ and δ^{13}C: from -16.6 to -17.5‰) were classified to the higher trophic level considering their feeding habits and the δ^{15}N ratios.

Investigation δ^{13}C data showed that *P. monodon* and *O. niloticus* deviated from the normal range of the rest of CE ecosystem dwelling organisms (Figure S1). The reason may

be because of their opportunistic ecological diversity where the juvenile *P. monodon* inhabits low salinity lagoons/ estuaries and *O. niloticus* resides in estuaries/lagoons and preferentially migrating to fresh water.

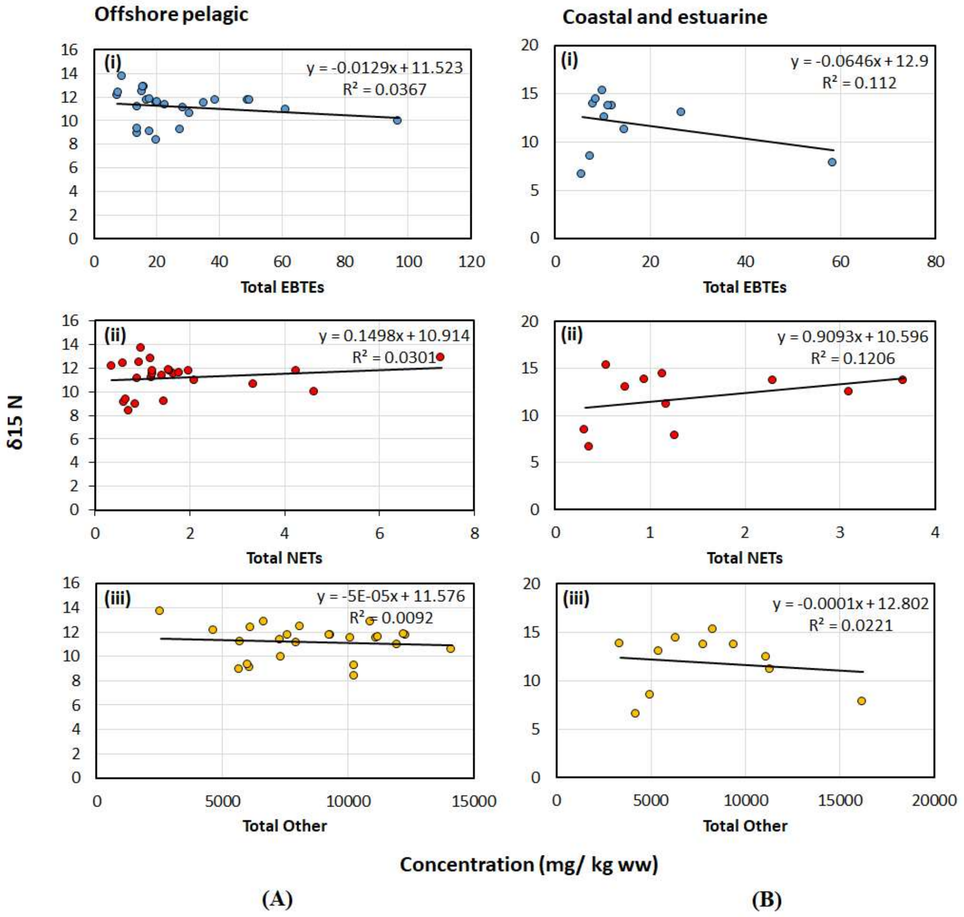

Figure S2. δ15 N verses concentrations of EBTEs, NETs and Other elements between off-shore pelagic (**A**) and coastal and estuarine (**B**) aquatic organisms collected in Sri Lanka.

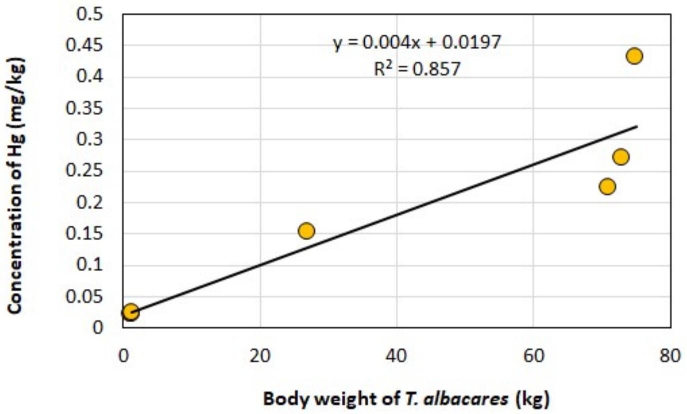

Figure S3. Relationship between the concentration of mercury (mg/ kg ww) and body weight of yellowfin tuna (*T. albacares*) collected in Sri Lanka.
